# *Notes from the Field*: Outbreak of *Escherichia coli* O157:H7 Infections Linked to Organic Walnuts — Washington and California, 2024

**DOI:** 10.15585/mmwr.mm7438a2

**Published:** 2025-11-27

**Authors:** Angelica L. Barrall, Laurie Stewart, Jeffrey Higa, Erin Jenkins, Brooke Whitney, Brandon Adcock, Anna Pickett, Bethan Swift, Peiman Aminabadi, Kenneth Zamora, Susan Shelton, Karen P. Neil, Laura Gieraltowski

**Affiliations:** ^1^Epidemic Intelligence Service, CDC; ^2^Division of Foodborne, Waterborne, and Environmental Diseases, National Center for Emerging and Zoonotic Infectious Diseases, CDC; ^3^Washington State Department of Health; ^4^California Department of Public Health; ^5^Coordinated Outbreak Response and Evaluation Network, Food and Drug Administration, College Park, Maryland; ^6^Riverside County Department of Public Health, Riverside, California; ^7^California Department of Food and Agriculture.

**Summary**

**What is already known about this topic?**

Outbreaks of Shiga toxin–producing *Escherichia coli* (STEC) O157 infections are most often associated with beef and leafy greens. Only one multistate outbreak of STEC O157 infections, in 2011, has been linked to tree nuts.

**What is added by this report?**

In 2024, investigators identified organic walnuts as the source of a multistate outbreak of STEC O157 infections, the first documented foodborne outbreak in the United States linked to walnuts.

**What are the implications for public health practice?**

Walnuts should be considered as a possible vehicle for STEC infections in future outbreaks.

Outbreaks of Shiga toxin–producing *Escherichia coli* (STEC) O157 infections are associated primarily with beef and fresh vegetables, particularly leafy greens* (*1*). Only one reported STEC O157 outbreak in the United States has been linked to tree nuts, specifically a 2011 outbreak in Michigan, Minnesota, and Wisconsin associated with in-shell hazelnuts^†^ (*2*). On March 25, 2024, the Washington State Department of Health alerted CDC to seven STEC O157 infections in Washington and California after determining that the isolates were highly genetically related by whole genome sequencing (WGS) (*3*).

## Investigation and Outcomes

On March 26, 2024, CDC began a multistate investigation using PulseNet, CDC’s national laboratory network for foodborne disease surveillance, to confirm the genetic relatedness of the initial seven infections and identify additional cases. A case was defined as infection with the outbreak strain or an isolate related within three allele differences of the outbreak strain by WGS, and illness onset during February 1–April 4, 2024, a date range selected to include all cases identified during the investigation. This activity was reviewed by CDC, deemed not research, and was conducted consistent with applicable federal law and CDC policy.^§^

A total of 13 cases from California (seven) and Washington (six) were identified (Figure). The median age of patients was 60 years (range = 6–84 years); 62% were female. Seven patients were hospitalized, two of whom developed hemolytic uremic syndrome, and none died.

**FIGURE:**
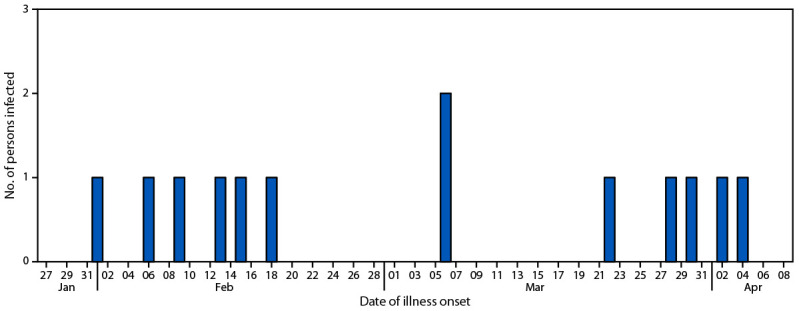
Number of persons infected with the outbreak strain of *Escherichia coli* O157 (N = 13), by date of illness onset — Washington and California, February 1–April 4, 2024

During initial interviews conducted by state partners, several patients reported shopping at food cooperatives (co-ops) and natural food stores in the week before becoming ill. Eleven patients who could be contacted were then interviewed with a questionnaire focused on fresh produce, nuts, and other products found at natural food stores (e.g., granola, seeds, and supplements). All 11 patients reported eating walnuts during the week preceding illness onset, and, as the investigation progressed, no other food item was found to be associated with illness. In comparison, 26% of healthy adults in FoodNet’s 2018–2019 Population Survey ate walnuts during the previous week (p<0.001; one-sample binomial test). All walnuts eaten by patients were specified during an interview or documented in purchase records at food co-ops or natural food stores as being “organic.” Ten patients purchased walnuts from bulk or self-service bins. Leftover walnuts from four patient homes (two each in California and Washington) were tested; one sample was positive for the gene encoding Shiga toxin by real-time polymerase chain reaction testing.

The Food and Drug Administration (FDA) and state partners analyzed records to trace the source of walnuts (which were purchased from nine different stores) eaten by the 11 patients who completed the focused questionnaire. Eight stores received organic walnut halves and pieces originating from two lots from the same processor, a sheller. A single grower supplied walnuts for both lots. FDA, the California Department of Public Health, and the California Department of Food and Agriculture performed inspections at the common processor and the common grower identified by the traceback investigation. Two product samples and 11 environmental samples were collected; none yielded STEC.

## Preliminary Conclusions and Actions

On April 27, 2024, the walnut processor recalled the two lots of walnut halves and pieces identified by the traceback investigation. On April 30, CDC and FDA advised the public to avoid consuming the recalled walnuts and provided a complete list of store names and locations that had received affected walnuts. The investigation was closed on June 25, 2024, when no additional illnesses meeting the case definition had been identified for several weeks, the environmental assessment had concluded, and the investigation team was confident that the contaminated walnuts were no longer available for purchase after the recall. Rapid detection, investigation, and product recall likely prevented additional illnesses from a product with a long shelf life. This outbreak demonstrates that walnuts can be contaminated with STEC and cause illness although the route of STEC contamination was not identified in this investigation. Producers of tree nuts should take steps to minimize the risk for bacterial contamination from the environment via multiple potential sources (e.g., water, soil, adjacent land use, and production environment) throughout growing, ground harvesting, hulling, shelling, and packing (*4*,*5*). Public health officials should consider walnuts as a possible vehicle for STEC infections in future outbreaks.

## Footnotes

*
Foodborne illness source attribution estimates for *Salmonella, Escherichia coli *O157, and *Listeria monocytogenes* — United States, 2021

†
National Outbreak Reporting System (NORS) Data | NORS | CDC

§ 45 C.F.R. part 46, 21 C.F.R. part 56; 42 U.S.C. Sect. 241(d); 5 U.S.C. Sect. 552a; 44 U.S.C. Sect. 3501 et seq.
